# Supplementary material for: Study of Polonium (210Po) Activity Concentration in Fruit Wines Derived from Different Locations in Poland
Source: Molecules. 2023 Jan 3;28(1):438. doi: 10.3390/molecules28010438 (PMC9824190; doi:10.3390/molecules28010438)
Supplement: Supplementary file 1 [file molecules-28-00438-s001.zip › molecules-2125931-supplementary.pdf]

Supplementary material

Study of Polonium (^{210}Po) Activity Concentration in Fruit Wines Derived from Different Locations in Poland

Paweł Rudnicki-Velasquez [1], Alicja Boryło [2], Marcin Kaczor [2], Jarosław Wieczorek [2] and Jarosława Rutkowska [1,*]

[1] Institute of Human Nutrition Sciences, Faculty of Human Nutrition, Warsaw University of Life Sciences (WULS-SGGW), Nowoursynowska St. 159c, 02-776 Warsaw, Poland
[2] Faculty of Chemistry, University of Gdańsk, Wita Stwosza Str. 63, 80-308 Gdańsk, Poland
* Correspondence: jaroslawa_rutkowska@sggw.edu.pl

Table S1. Characteristic of fruit wine samples.

Sample ID	Type of fruits used for wine production	Colour and type of wine
**Wines from Warmian-Masurian voivodeship**		
W1	multi-fruit (cherry, yeast)	red; semi-dry
W2	multi-fruit	red; sweet
W3	gean	red; semi-sweet
W4	cherry	red; semi-sweet
W5	apple	white; dry
W6	multi-fruit (cherry, raspberry)	red; semi-sweet
W7	multi-fruit (cherry, raspberry)	red; semi-sweet
W8	multi-fruit (apple, apricot)	white; semi-sweet
W9	multi-fruit (black and red currants)	red; dry
W10	multi-fruit (black and red currant, raspberry)	red; semi-dry
W11	multi-fruit (cherry, raspberry, black currant)	red; sweet
W12	multi-fruit (cherry, raspberry, currant)	red; semi-dry
W13	multi-fruit (black and red currant, apple, gooseberry, chokeberry)	red; semi-sweet
W14	multi-fruit (black and red currants, apple, gooseberry, chokeberry)	red; semi-sweet
W15	apple	white; semi-sweet
W16	multi-fruit (raspberry, cherry, herbs)	red; dry
W17	apple	white; sweet
W18	multi-fruit (raspberry, cherry, plum)	red; dry
**Wines from Podlaskie voivodeship**		
P1	apple	white; semi-dry
P2	apple	white; semi-sweet
P3	multi-fruit (chokeberry, gooseberry)	white; dry
P4	cherry	red; semi-sweet
P5	cherry	red; semi-sweet
P6	cherry	red; dry
P7	cherry	red; semi-sweet
P8	black currant	red; semi-sweet
P9	cherry	red; dry
P10	multi-fruit (cherry, yeast)	red; semi-dry
P11	raspberry	red; semi-sweet
P12	multi-fruit (red currant, apple)	red; semi-dry
P13	multi-fruit (apple, pear)	white; sweet
P14	chokeberry	white; semi-sweet
P15	quince	white; dry
P16	strawberry	pink; dry
P17	multi-fruit (raspberry, strawberry, chokeberry)	red; semi-dry

P18	multi-fruit (apple, gooseberry)	white; dry
P19	multi-fruit (apple, currant)	white; sweet
P20	multi-fruit (cherry, bread, yeast)	red; semi-sweet
**Wines from Małopolskie voivodeship**		
M1	red currant	red; semi-dry
M2	chokeberry	red; semi-dry
M3	black currant	red; dry
M4	multi-fruit (pear, apple)	white; dry
M5	red currant	red; semi-dry
M6	mint	white; dry
M7	multi-fruit (apple, apricot)	white; sweet
M8	multi-fruit (black and red currant)	red; semi-sweet
M9	multi-fruit (plum, raspberry)	dry; semi-sweet
M10	multi-fruit (cherry, pear, quince)	red; semi-dry
M11	gooseberry	white; dry
M12	multi-fruit (apricot, quince, red currants)	red; dry
M13	multi-fruit (apricot, gooseberry)	white; semi-sweet
M14	multi-fruit (apricot, pear, quince)	white; semi-dry
M15	multi-fruit (apricot, cherry)	red; semi-sweet
M16	multi-fruit (apple, quince)	white; semi-sweet
M17	multi-fruit (pear, quince, mint)	red; semi-dry
M18	multi-fruit (apricot, pear)	white; semi-sweet
M19	multi-fruit (pear, quince, raspberry)	red; semi-dry
M20	multi-fruit (apple, quince, gooseberry)	red; semi-dry
M21	multi-fruit (raspberry, cherry, plum)	red; dry
M22	multi-fruit (raspberry, plum, mint)	red; dry
M23	multi-fruit (raspberry, mint)	white; semi-dry
M24	multi-fruit (apricot, gooseberry)	white; dry
M25	multi-fruit (gooseberry, apricot)	red; dry
**Wines from Lubelskie voivodeship**		
L1	cherry	red; semi-dry
L2	apple	white; dry
L3	chokeberry	red; semi-dry
L4	chokeberry	red; semi-dry
L5	cherry	white; dry
L6	pear	white, dry
L7	multi-fruit (cherry, plum)	red; semi-dry
L8	pear	pink; sweet
L9	multi-fruit (cherry, gean)	white; semi-dry
L10	red currant	red; semi-dry
L11	multi-fruit (currant, plum)	red; dry
L12	multi-fruit (strawberry, chokeberry)	red; semi-dry
L13	multi-fruit (raspberry, currant)	red; semi-dry
L14	multi-fruit (strawberry, raspberry)	pink; semi-sweet
L15	multi-fruit (apricot, apple)	white; semi-sweet

L16	multi-fruit (pear, quince, raspberry)	red; dry
L17	multi-fruit (apricot, mint)	white; semi-sweet
L18	multi-fruit (apricot, quince)	red; dry
L19	raspberry	red; semi-sweet
L20	multi-fruit (apricot, apple)	white; semi-sweet
L21	multi-fruit (black currant, raspberry)	red; dry
L22	raspberry	pink; sweet
L23	multi-fruit (currant, gooseberry)	white; semi-dry
